# Development and characterization of LipoCatch: a bacterial lipoprotein-based biomaterial that self-assembles into nanostructures

**DOI:** 10.1039/d6na00554c

**Published:** 2026-07-09

**Authors:** Francesca A. Starvaggi, Claire J. Stewart, Marc A. Arslanian, Matthew A. Treviño, Scott A. Weston, Naima G. Sharaf

**Affiliations:** a Department of Biology, Stanford University Stanford CA 94305 USA ngsharaf@stanford.edu; b Department of Chemistry, Stanford University Stanford CA 94305 USA

## Abstract

Genetically encoded nanomaterials enable the control of molecular composition and function, yet the use of the biosynthetic bacterial lipidation pathway to build hybrid protein/lipid nanostructures has not been reported. Here, we designed a versatile bacterial lipoprotein, named LipoCatch, for modular nanostructure formation. Lipidation was achieved by appending a signal peptide to the protein-encoding gene of SpyCatcher from the SpyCatcher/SpyTag protein/peptide binding pair. This protein was biosynthetically produced in *E. coli* and purified in the presence of detergent. LC-MS, SEC, DLS, and TEM confirmed site-specific lipidation and formation of nanoparticles with an average diameter of 13 nm. We show that LipoCatch supports two orthogonal functionalization strategies: (1) covalent modification through the SpyCatcher/SpyTag system and (2) the non-covalent incorporation of phospholipids that permits the tuning of particle size and compositions. Finally, stability studies show LipoCatch and hybrid LipoCatch/phospholipid nanostructures are tolerant to lyophilization, in contrast to phospholipid-only liposomes. Together, this proof-of-principle study establishes an engineered bacterial lipoprotein, LipoCatch, as a genetically encoded platform for building customizable bacterial lipoprotein-based biomaterials.

## Abstract 2

Genetically encoded nanomaterials enable the control of molecular composition and function, yet the use of the biosynthetic bacterial lipidation pathway to build hybrid protein/lipid nanostructures has not been reported.

## Graphical abstract

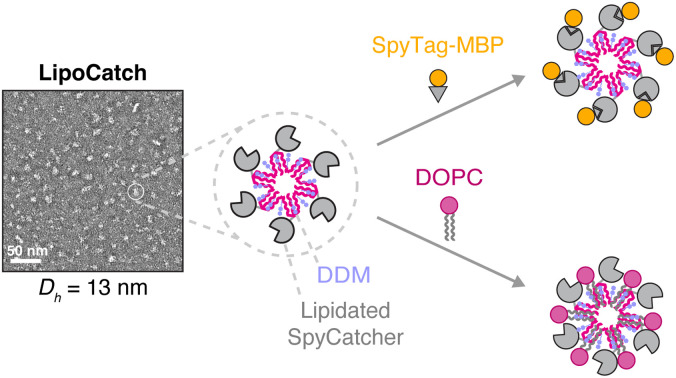

## Introduction

Bacterial lipoproteins are characterized by a covalently attached lipid moiety that drives their spontaneous organization into nanostructures when removed from their native membranes.^1,2^ This class of proteins is structurally similar to lipopeptide amphiphiles, which form diverse nanostructures useful as delivery vehicles for therapeutics and vaccine platforms.^3,4^ Both combine an amino acid stretch to impart molecular functions and a hydrophobic lipid moiety to drive self-assembly. However, bacterial lipoproteins and lipopeptide amphiphiles have several key differences. Peptide amphiphiles contain short amino acid stretches, whereas bacterial lipoproteins feature larger, structured globular protein domains. Furthermore, peptide amphiphiles are typically produced using chemical synthesis,^3^ while bacterial lipoproteins are produced biosynthetically by exploiting the bacterium's endogenous lipoprotein biosynthesis pathway. This pathway involves the post-translational lipidation of the protein through site-specific attachment of acyl chains to an N-terminal cysteine residue.^5^ A key advantage of bacterial lipoproteins is their genetically encoded protein domain that enables precise molecular engineering.^6,7^ This feature could enable rapid prototyping and scalable production through simple modification of the protein-encoding gene in the expression vector. While lipopeptides are versatile, bacterial lipoproteins have a similar ability to self-assemble into nanostructures, with a potentially broadened scope by engineering the protein domain for specific purposes (*e.g.* targeting motif, antigen, enzymatic domain). Despite their potential, bacterial lipoproteins have not been previously reported as building blocks for nanostructures.

To establish bacterial lipoproteins as versatile building blocks for biomaterials, we designed LipoCatch: a genetic fusion of a lipoprotein signal peptide and the protein SpyCatcher. SpyCatcher was chosen for its ability to form an irreversible isopeptide bond with its peptide binding pair SpyTag.^8^ We envisioned this system would allow facile attachment of SpyTagged proteins to the LipoCatch nanostructure. LipoCatch was successfully recombinantly expressed and its lipidation state was characterized by liquid chromatography mass spectrometry (LC-MS). Nanostructures formed by detergent-solubilized LipoCatch were characterized using size exclusion chromatography (SEC), dynamic light scattering (DLS), and negative stain transmission electron microscopy (TEM). Since the interactions of lipoprotein nanostructures with other molecules are not well characterized, we examined two modification strategies. First, we showed covalent functionalization using SpyTagged proteins (SpyTag-GFP and SpyTag-MBP). Second, we developed a protocol for non-covalent association of LipoCatch with exogenous phospholipids. Finally, we assessed the stability of the resulting nanostructures to lyophilization. Together, these experiments provide a foundation for future development of bacterial lipoprotein-based biomaterials.

## Results

### Production and characterization of detergent-solubilized LipoCatch nanostructures

**Fig. 1:**
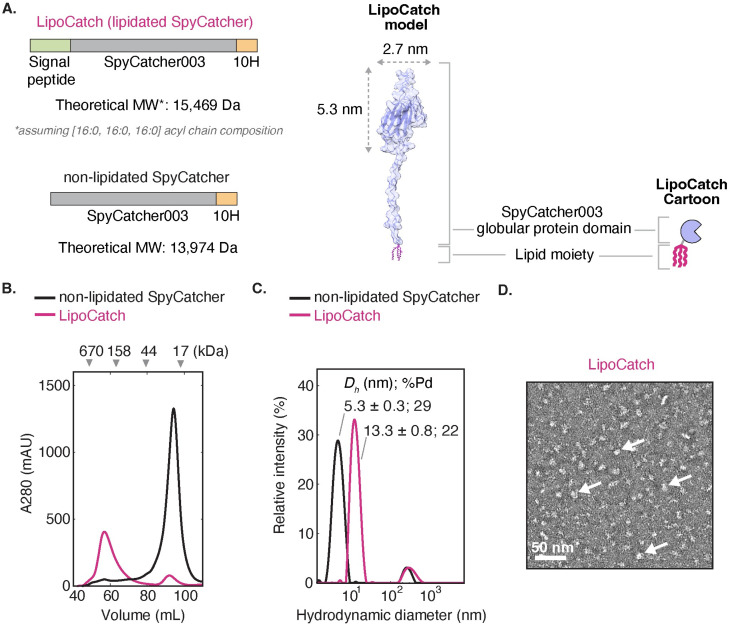
Production and characterization of LipoCatch and non-lipidated SpyCatcher. (A) Schematic of the protein sequences of LipoCatch and non-lipidated SpyCatcher. The signal peptide of LipoNmMetQ, SpyCatcher003 and the decahistidine tag, are shown as green, gray and orange rectangles, respectively. (B) SEC elution profiles of LipoCatch and of non-lipidated SpyCatcher. Peak elution volumes of gel filtration standards are indicated by gray triangles. (C) DLS traces of LipoCatch and non-lipidated SpyCatcher. The mean *D*_h_ (nm) and %Pd are indicated next to the major peak (*n* = 3). (D) Representative negatively stained TEM micrograph of LipoCatch nanostructures. White arrows indicate individual nanostructures. Scale bar: 50 nm.

Previous research found that recombinantly overexpressed and purified lipoproteins self-assembled into nanostructures in solution.^1,2^ However, the rules governing their association with other molecules remain poorly understood. To explore the potential of bacterial lipoproteins as components of nanostructures, we set out to engineer a versatile lipoprotein that would allow us to explore covalent and non-covalent interactions with proteins and phospholipids. For this study, we chose to leverage the SpyCatcher/SpyTag system.^8^ Since the SpyCatcher protein is not natively lipidated, we genetically fused the signal peptide of the lipoprotein NmMetQ from *Neisseria meningitidis* (LipoNmMetQ, Uniprot ID: Q7DD63) at the N-terminus of the SpyCatcher003 protein-encoding gene (Fig. S1). We chose the signal peptide of LipoNmMetQ based on our previous work which showed that recombinant overexpression of the full-length NmMetQ protein-encoding gene resulted in the production of a triacylated LipoNmMetQ.^1^ For purification purposes, a decahistidine tag (10H) was fused at the C-terminus (Fig. 1A). We call this protein LipoCatch. As a control, we designed a construct encoding SpyCatcher lacking the signal peptide, yielding a non-lipidated form of SpyCatcher. Both proteins were then recombinantly produced in *Escherichia coli* (*E. coli*), and purified using affinity chromatography followed by SEC in the presence of 0.05% w/v *n*-Dodecyl-β-d-Maltoside (DDM).

As expected, the SEC elution trace of the control protein, non-lipidated SpyCatcher, revealed a single peak with an elution volume of 94 mL (Fig. 1B, black trace). Comparing this elution volume to gel chromatography standards (Fig. 1B, gray triangles), nonlipidated SpyCatcher eluted in a single peak consistent with its monomeric theoretical mass of 13 974 Da. In contrast, the SEC elution trace for LipoCatch revealed two peaks: a major peak at 57 mL and a minor peak at 92 mL (Fig. 1B, magenta trace). The elution volume of the major peak of LipoCatch corresponds to a much larger molecular weight than the theoretical molecular weight of monomeric LipoCatch (15 469 Da) based on comparison to the gel filtration standards, while the minor peak eluted similarly to non-lipidated SpyCatcher. Together, these results show that fusion of the signal peptide encoding sequence from LipoNmMetQ to the SpyCatcher protein-encoding gene produces a higher molecular weight protein population than that of the monomeric lipoprotein alone, suggesting that LipoCatch self-assembles or possibly associates with DDM micelles (∼70 kDa)^9^ when analyzed by SEC.

To determine the lipidation state of LipoCatch, we analyzed the major and minor SEC peaks using LC-MS (Fig. S2). We first estimated the theoretical molecular weights of the isoforms created at each step of the lipoprotein maturation pathway (Fig. S2A). This estimation utilized the fatty acid composition of *E. coli* lipids, where palmitic acid (16 : 0) and oleic acid (18 : 1) are the most common.^10,11^ Analysis of the SEC major peak (57 mL elution volume) showed a total ion chromatogram with two peaks at retention times of 7.56 and 10.11 min, revealing deconvoluted masses of 16 643 Da, and 15 468 Da/15 486 Da/15 495 Da, respectively (Fig. S2B). The larger mass at a retention time of 7.56 min (measured mass = 16 643 Da) corresponds well to the theoretical mass of non-lipidated LipoCatch, containing an intact signal peptide (theoretical mass = 16 644 Da). Closer inspection of the deconvoluted masses at a retention time of 10.11 min (measured masses = 15 468/15 486/15 495 Da) closely align with the theoretical masses of SpyCatcher modified at cysteine 20 with a triacyl chain composition of [16 : 0, 16 : 0, 16 : 0] (theoretical mass 15 469 Da), [16 : 0, 17 : 1 (cyclo), 16 : 0] (theoretical mass = 15 486 Da), and [16 : 0, 18 : 1, 16 : 0] (theoretical mass = 15 495 Da). The numbers within the square brackets denote, in order, the total number of carbons and the number of unsaturation elements per acyl chain, with cyclopropane rings explicitly distinguished from C=C double bonds. Together, these data suggest that LipoCatch is a mixture of two proteins: an immature protein with an intact signal peptide and a heterogeneously lipidated protein with three acyl chains.

To confirm whether LipoCatch forms nanostructures, we analyzed the SEC major peak using DLS in comparison to non-lipidated SpyCatcher. As expected, DLS analysis of non-lipidated SpyCatcher revealed a single peak with an average hydrodynamic diameter (*D*_h_) of 5.3 ± 0.3 nm (Fig. 1C, black trace). The size of LipoCatch was larger with a *D*_h_ of 13.3 ± 0.8 nm, suggesting that LipoCatch forms nanostructures larger than the monomeric protein (Fig. 1C, pink trace). TEM micrographs of LipoCatch revealed irregularly shaped, micelle-like nanostructures (Fig. 1D). Together, our results show that LipoCatch forms nanostructures in solution that are larger than monomeric, non-lipidated SpyCatcher.

### LipoCatch can be covalently functionalized with SpyTagged proteins

**Fig. 2:**
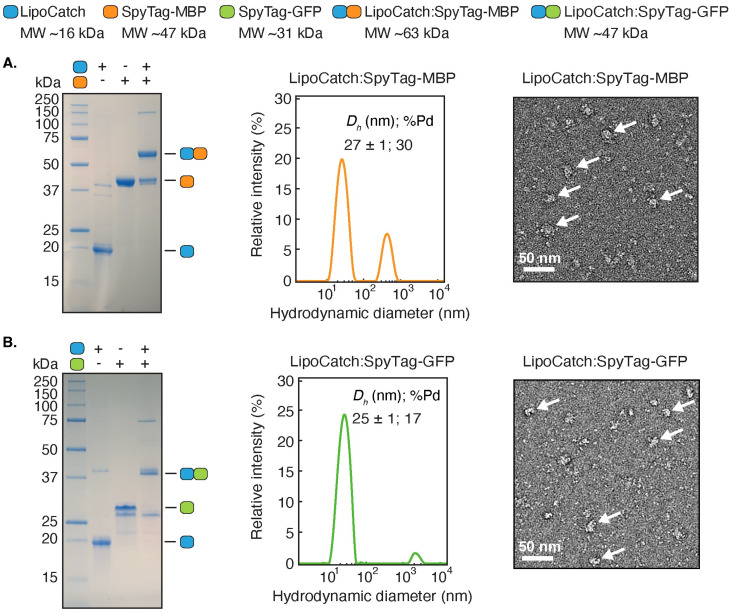
Formation and characterization of LipoCatch nanostructures functionalized with SpyTagged proteins. Analysis of the coupling of (A) LipoCatch : SpyTag-MBP and (B) LipoCatch : SpyTag-GFP using SDS-PAGE (left), DLS (middle), and TEM (right). The mean *D*_h_ (nm) and %Pd are indicated next to the major peak (*n* = 3). Color coding of LipoCatch, SpyTag-MBP, and SpyTag-GFP samples with their approximate theoretical molecular weights (top).

To ensure that the appended lipid moiety did not impair SpyCatcher's ability to conjugate to SpyTagged proteins, we assessed conjugation of LipoCatch to SpyTag-MBP and SpyTag-GFP. These proteins were selected because previous studies have demonstrated their ability to form a covalent bond with SpyCatcher.^12,13^ As expected, SDS-polyacrylamide gel electrophoresis (SDS-PAGE) analysis of LipoCatch alone (MW ∼16 kDa) revealed a single band below 20 kDa. When LipoCatch was mixed with SpyTag-MBP (MW ∼47 kDa) at a 1 : 1 molar ratio, SDS-PAGE analysis showed a shift in molecular weight above 50 kDa, indicating the formation of a covalent bond between LipoCatch and SpyTag-MBP (MW ∼63 kDa) (Fig. 2A, left panel). Similarly, after mixing LipoCatch with SpyTag-GFP (MW ∼31 kDa) at a 1 : 1 molar ratio, SDS-PAGE revealed a shift in molecular weight above 37 kDa indicating covalent bond formation between LipoCatch and SpyTag-GFP (MW ∼47 kDa) (Fig. 2B, left panel). Together, these data show that LipoCatch retains its ability to conjugate to SpyTagged proteins.

Nanostructure size is a key determinant of how nanoparticles interact with biological systems, including cellular barriers, tissue penetration, and biodistribution.^14^ Therefore, we investigated whether the conjugation with SpyTagged proteins compromises nanostructure integrity or induces measurable changes in size and morphology. For these experiments, LipoCatch : SpyTag-MBP and LipoCatch : SpyTag-GFP conjugates were analyzed by DLS and TEM. DLS analysis of LipoCatch : SpyTag-MBP revealed a major peak with a *D*_h_ of 27 ± 1 nm and a %Pd of 30% (Fig. 2A, orange trace, middle panel). Similarly, DLS analysis of LipoCatch : SpyTag-GFP showed a major peak with a *D*_h_ of 25 ± 1 nm, but a lower %Pd of 17% (Fig. 2B, green trace, middle panel). A minor peak of larger size was also observed in DLS traces of both LipoCatch : SpyTag-MBP and LipoCatch : SpyTag-GFP, possibly representing aggregated protein; the average size of the minor peaks was not calculated due to irreproducibility. The higher %Pd observed for LipoCatch : SpyTag-MBP compared to LipoCatch : SpyTag-GFP could be attributed to several factors, including the higher molecular weight of SpyTag-MBP, the overall shape of the protein domain, or differences in surface charge distribution. Both LipoCatch : SpyTag-MBP and LipoCatch : SpyTag-GFP showed a shift to a larger size compared to LipoCatch alone (*D*_h_ = 13.3 ± 0.8 nm), suggesting that nanostructure integrity is preserved. TEM analysis further confirmed that both LipoCatch : SpyTag-MBP and LipoCatch : SpyTag-GFP formed irregularly shaped, micelle-like nanostructures (Fig. 2A, right panel and Fig. 2B, right panel, respectively). Together, these results show that conjugation of LipoCatch to SpyTag-MBP or SpyTag-GFP increases nanostructure size, while preserving the formation of intact, micelle-like nanostructures.

### LipoCatch forms nanostructures with DOPC

After establishing that LipoCatch nanostructures can remain intact when covalently attached to select SpyTagged proteins, we next asked whether they can also be modified non-covalently. Since LipoCatch contains a lipid moiety, we reasoned that it could be formulated with exogenously supplied phospholipids. Specifically, we asked whether adding phospholipids compromises nanostructure integrity or induces distinct nanostructure morphologies, such as liposome formation, given the intrinsic propensity of phospholipids to self-assemble into vesicular structures. To test this, we investigated the assembly of LipoCatch with the phospholipid 1,2-dioleoyl-*sn*-glycero-3-phosphocholine (DOPC).

Standard liposome preparations often involve dissolving lipids in an organic solvent, forming a lipid thin film by solvent removal, hydrating the film, and agitating the sample to produce large, multilamellar vesicles (Fig. S3A). Sizing procedures such as extrusion or sonication are subsequently employed to generate unilamellar vesicles.^15^ For the LipoCatch/DOPC formulations, we developed a protocol that maintains this general workflow but incorporates LipoCatch during the hydration step (Fig. S3B). The addition of the protein solution after thin film formation ensures that LipoCatch avoids contact with the organic solvent, allowing it to retain its native three-dimensional structure. The mixture was then subjected to centrifugal ultrafiltration to promote LipoCatch-phospholipid association. No additional sizing procedures were performed. As a control, a DOPC alone sample was produced using the same procedure.

**Fig. 3:**
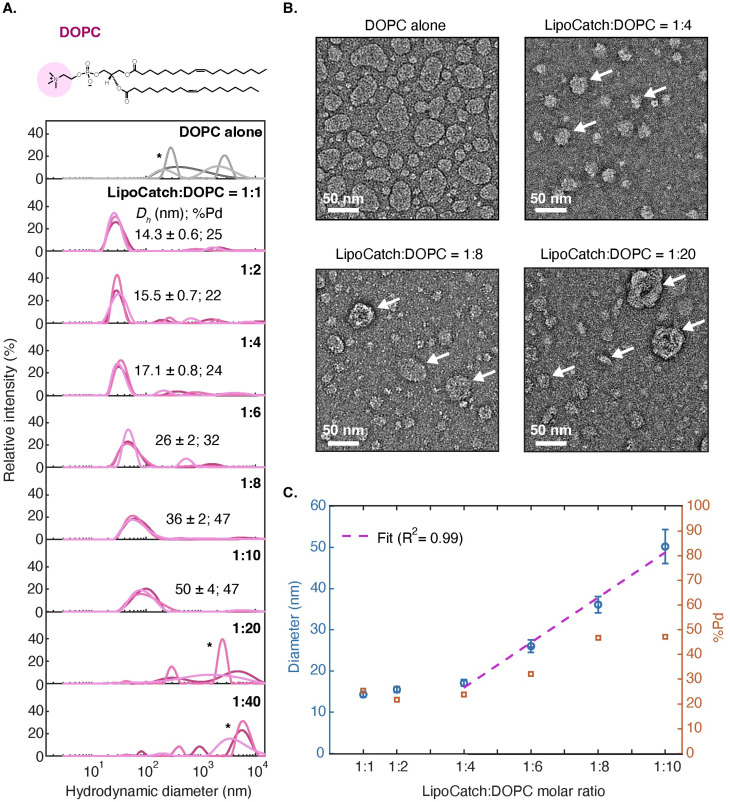
Characterization of LipoCatch/DOPC nanostructures. (A) DLS measurements of DOPC alone and LipoCatch/DOPC nanostructures. DOPC alone was formulated at 0.3624 mM with 0.05% DDM. LipoCatch : DOPC samples were formulated at 0.092 mM LipoCatch. The mean *D*_h_ (nm) and %*P*_d_ are indicated next to the major peak (*n* = 3). Compositions with irreproducible size distributions are indicated with an asterisk (*). (B) Representative TEM micrographs of DOPC alone and LipoCatch/DOPC nanostructures with molar ratios of LipoCatch : DOPC = 1 : 4, 1 : 8, and 1 : 20. White arrows indicate individual nanostructures. Scale bar = 50 nm. (C) Linear fit of nanostructure diameter and %Pd *vs.* LipoCatch : DOPC molar ratio.

To analyze the size and morphology of the LipoCatch/DOPC samples, we used DLS and TEM. It is known that detergents can associate with phospholipids. Therefore, a DOPC control with detergent (final DDM concentration = 0.05%) was prepared similarly to LipoCatch to ensure a more direct comparison. As expected, the DOPC alone sample resulted in multimodal DLS traces with a broad distribution of *D*_h_ (Fig. 3A, gray traces). Consistent with these results, TEM revealed a heterogeneous population of vesicles, indicative of liposome-like structures that have not undergone sizing procedures (Fig. 3B, top left panel). Next, we formulated LipoCatch/DOPC samples by fixing the concentration of LipoCatch and increasing the DOPC molar equivalents from 1 to 40 (Fig. 3A, pink traces). DLS analysis of LipoCatch/DOPC (1 : 1) nanostructures revealed a single monomodal peak with a *D*_h_ of 14.3 ± 0.6 nm (Fig. 3A, pink traces, second panel). The *D*_h_ of LipoCatch/DOPC nanostructures at molar ratios of 1 : 2, 1 : 4, 1 : 6, 1 : 8, and 1 : 10 were determined to be 15.5 ± 0.7 nm, 17.1 ± 0.8 nm, 26 ± 2 nm, 36 ± 2 nm, and 50 ± 4 nm, respectively, each derived from monomodal DLS peaks. Notably, the *D*_h_ of LipoCatch/DOPC nanostructures increased linearly from molar ratios of 1 : 4 to 1 : 10 (Fig. 3C, left axis). The %Pd also increased between molar ratios of 1 : 4 and 1 : 10, from 24% to 47%, respectively (Fig. 3C, right axis). TEM analysis confirmed the homogeneity of LipoCatch/DOPC (1 : 4) nanostructures and a slightly more heterogeneous population for LipoCatch/DOPC (1 : 8) (Fig. 3B, top right panel and bottom left panel, respectively). In contrast, DLS analysis of LipoCatch/DOPC (1 : 20) and LipoCatch/DOPC (1 : 40) samples revealed multimodal traces overlapping in size with the DOPC alone control (Fig. 3A, bottom two panels). This indicates a heterogeneous sample composition, potentially containing DOPC only assemblies. Confirming the DLS results, a TEM micrograph of LipoCatch/DOPC (1 : 20) revealed a more heterogeneous mixture of nanostructures containing large, irregularly aggregated assemblies. Together, these data define a compositional window (1 : 1–1 : 10) in which LipoCatch/DOPC nanostructures exhibit predictable, molar-ratio-dependent increases in hydrodynamic diameter and %Pd, thereby establishing a protocol for generating size-tunable assemblies.

### LipoCatch forms nanostructures with phospholipids of various headgroups

**Fig. 4:**
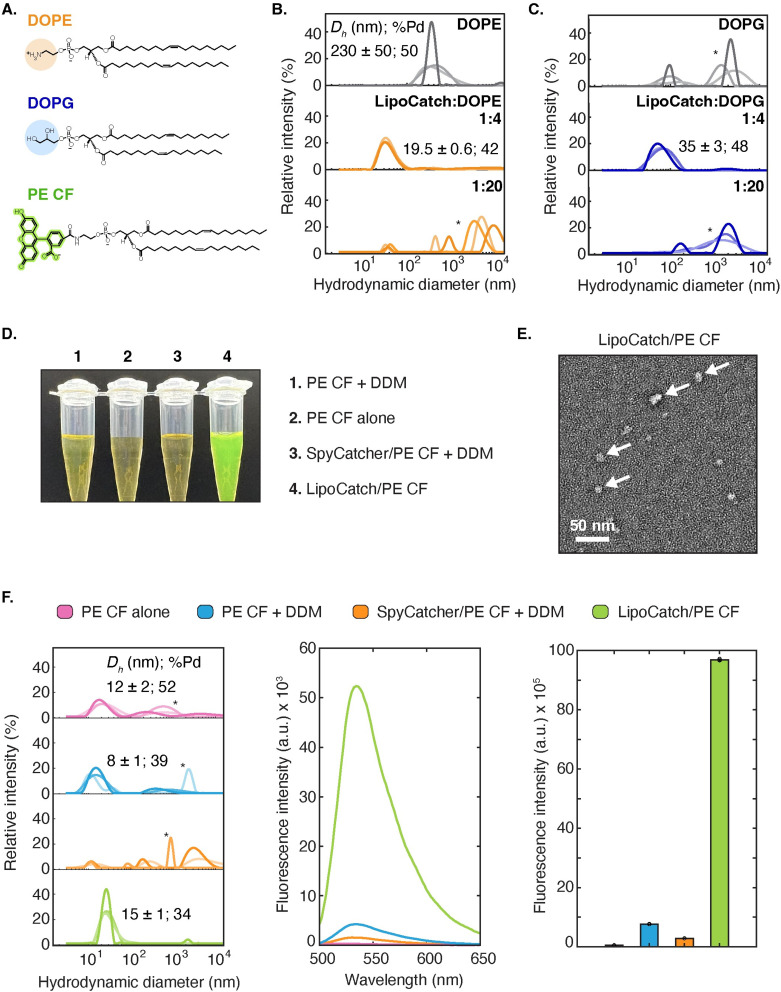
Characterization of various LipoCatch/phospholipid nanostructures. (A) Chemical structures of DOPE (orange), DOPG (blue), and PE CF (green). DLS measurements of (B) LipoCatch/DOPE and (C) LipoCatch/DOPG nanostructures. (D) Photos of PE CF samples. (E) Representative TEM micrograph of LipoCatch/PE CF (1 : 1 molar ratio). White arrows indicate individual nanostructures. Scale bar = 50 nm. (F) Color coding for PE CF samples (top). DLS measurements (left), fluorescence spectra (middle), and fluorescence emission intensities (excitation/emission = 485/528 nm) (right). The mean *D*_h_ (nm) and %Pd are indicated next to the major peak (*n* = 3). Compositions with irreproducible size distributions are indicated with an asterisk (*).

To examine the effect of phospholipid headgroup structure on assembly with LipoCatch, we first selected two additional phospholipids with distinct headgroups but identical acyl chain structures to DOPC: 1,2-dioleoyl-*sn*-glycero-3-phosphoethanolamine (DOPE; neutral lipid) and 1,2-dioleoyl-*sn*-glycero-3-phosphoglycerol (DOPG; negatively charged lipid) (Fig. 4A). While either the phospholipid headgroup or acyl chains could be varied, to enable formulation at room temperature, phospholipids with oleic acid (18 : 1) acyl chains were chosen due to their low phase transition temperatures (*T*_m_) of −17 °C, −16 °C, and −18 °C for DOPC, DOPE, and DOPG, respectively.^16^ PC, PE, and PG headgroups were chosen due to their low cost and commercial availability.

LipoCatch/DOPE and LipoCatch/DOPG nanostructures were prepared with LipoCatch : phospholipid molar ratios of 1 : 4 or 1 : 20, using the same modified thin-film hydration procedure as described for LipoCatch/DOPC preparation. These molar ratios were selected because they led to distinctly different assembly characteristics of LipoCatch/DOPC – homogeneous nanostructures at 1 : 4 and heterogeneous nanostructures at 1 : 20. As controls, we used the same procedure to produce DOPE-only and DOPG-only structures, which includes the addition of DDM in the hydration step. DLS analysis of DOPE-only structures showed monomodal peaks with an average diameter of 230 ± 50 nm, while DLS analysis of DOPG-only structures showed multimodal traces indicating a wide distribution of sizes, as expected for phospholipid assemblies that have not undergone sizing procedures (Fig. 4B, gray traces and Fig. 4C, gray traces, respectively). In contrast, DLS analysis of LipoCatch/DOPE (1 : 4) nanostructures showed monomodal peaks with a *D*_h_ of 19.5 ± 0.6 nm and a %Pd of 42% (Fig. 4B, orange traces, middle panel). Similarly, DLS analysis of LipoCatch/DOPG (1 : 4) nanostructures showed monomodal peaks with a *D*_h_ of 35 ± 3 nm and a %Pd of 48% (Fig. 4C, blue traces, middle panel). This represents a size increase of 15.5 nm and 17.9 nm compared to LipoCatch/DOPE and LipoCatch/DOPC assemblies, respectively. Similar to the LipoCatch/DOPC experiments, DLS analysis of LipoCatch : DOPE (1 : 20) or LipoCatch : DOPG (1 : 20) revealed multimodal traces overlapping in size with the DOPG alone control (Fig. 4B, orange traces, bottom panel and Fig. 4C, blue traces, bottom panel, respectively). This indicates heterogeneous populations of nanostructures, possibly including phospholipid only assemblies. Together, these data reveal that regardless of whether the phospholipid headgroup is choline, ethanolamine, or glycerol, these phospholipids associate with LipoCatch at a 1 : 4 molar ratio to form homogeneous nanostructures, suggesting that there is no detectable trend for assembly with LipoCatch based on phospholipid headgroup structure or charge.

Next, we explored the assembly of LipoCatch with a non-natural phospholipid to further assess the scope of lipids capable of non-covalent association with the LipoCatch nanostructure. For this study we chose a phospholipid with the same acyl chain composition as DOPC, but containing a non-natural carboxyfluorescein headgroup, 1,2-dioleoyl-sn-glycero-3-phosphoethanolamine-*N*-(carboxyfluorescein) (PE CF). This molecule was chosen because it is commercially available and fluorescent, enabling sensitive detection. We prepared samples of LipoCatch/PE CF, non-lipidated SpyCatcher/PE CF, and PE CF alone. The non-lipidated SpyCatcher/PE CF control sample was prepared with DDM, and PE CF alone samples were prepared with and without DDM. All samples were initially prepared using the thin-film hydration protocol developed in this work at a PE CF and LipoCatch concentration of 50 µM, followed by an adjustment to the same initial volume after centrifugation.

DLS analysis of PE CF without or with DDM revealed major peaks with a *D*_h_ of 12 ± 2 nm or 8 ± 1 nm, respectively, suggesting that PE CF aggregates in solution (Fig. 4F, pink traces, left panel, and Fig. 4F, blue traces, left panel, respectively). Analysis of another control sample, non-lipidated SpyCatcher/PE CF + DDM at a 1 : 1 molar ratio revealed irreproducible, multimodal DLS traces indicating a broad distribution of *D*_h_ (Fig. 4F, orange traces, left panel). These data suggest that the protein domain of non-lipidated SpyCatcher alone, in the presence of DDM, is not sufficient to promote the formation of well-defined, monodisperse assemblies. In contrast, DLS analysis of LipoCatch/PE CF at a 1 : 1 molar ratio showed monomodal traces with an average *D*_h_ of 15 ± 1 nm and a %Pd of 34%, suggesting the formation of LipoCatch/PE CF nanostructures with uniform size (Fig. 4F, green traces, left panel). TEM analysis of a LipoCatch/PE CF sample revealed micelle-like nanostructures also of homogeneous size (Fig. 4E). Notably, a striking change in fluorescence was visually detected which, when quantified, revealed a 225-fold increase in fluorescence emission at 528 nm of LipoCatch/PE CF compared to PE CF alone without DDM (Fig. 4F, green bar, right panel and Fig. 4F, pink bar, right panel, respectively). The strong fluorescence of the LipoCatch/PE CF sample can also be seen visually compared to the control samples, suggesting that the presence of LipoCatch influences the properties of the final formulation compared to PE CF alone, PE CF + DDM, or SpyCatcher/PE CF + DDM (Fig. 4D). The change in fluorescence observed in the presence of LipoCatch could possibly be due to reduced self quenching of PE CF upon co-assembly with LipoCatch nanostructures. Interestingly, we also observed a red shift in the fluorescence emission maximum of LipoCatch/PE CF to 534 nm in comparison to PE CF alone (522 nm). While the molecular basis of this shift remains unclear, the observed spectral changes suggest that the local environment of PE CF is altered upon association with LipoCatch, possibly due to altered fluorophore aggregation.

### Stability

**Fig. 5:**
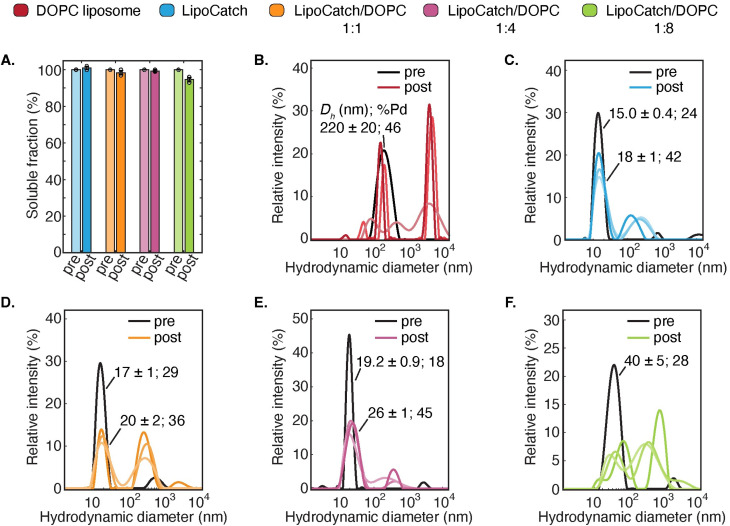
Stability of nanostructures to lyophilization. (A) Protein soluble fraction pre and post lyophilization and reconstitution in the same volume (mean ± 1 s.d., *n* = 3). DLS measurements of (B) DOPC-only liposomes produced using thin film hydration and extrusion, (C) LipoCatch, (D) LipoCatch/DOPC (1 : 1), (E) LipoCatch/DOPC (1 : 4), and (F) LipoCatch/DOPC (1 : 8) pre (black line) and post (colored line) lyophilization and reconstitution in the same volume. The mean *D*_h_ (nm) and %Pd are indicated next to the major peak (*n* = 3). Color coding for DOPC, LipoCatch, and LipoCatch/DOPC nanostructures (top).

Lyophilization is a well-established method used to improve nanoparticle stability and extend shelf life.^17^ However, this process is problematic for certain nanoparticle systems, such as liposome-based formulations that often exhibit structural instability. Such systems therefore often require cryoprotectants or carefully optimized formulation strategies to ensure their stability during freezing, drying, and rehydration.^18^ To assess LipoCatch stability upon lyophilization, we examined the soluble fraction of protein after lyophilization and rehydration as a measure of percent aggregation using gel densitometry. LipoCatch remained 100% soluble after lyophilization and rehydration, while LipoCatch/DOPC (1 : 1), LipoCatch/DOPC (1 : 4), and LipoCatch/DOPC (1 : 8) remained 98%, 99%, and 95% soluble, respectively (Fig. 5A). This data shows that LipoCatch alone and within LipoCatch/DOPC assemblies retain high solubility following lyophilization and rehydration.

After establishing that LipoCatch remains in solution after lyophilization and rehydration, we next evaluated the effects of lyophilization on nanostructure integrity. For these studies, we used DLS to characterize their hydrodynamic diameter before and after lyophilization and compared the results to a DOPC liposome control. Unlike previous DOPC controls, this liposome control was not prepared using DDM but instead prepared traditionally using thin-film hydration followed by sizing procedures. DLS analysis revealed a single monomodal peak with a *D*_h_ of 220 ± 20 nm prior to lyophilization, whereas rehydration resulted in irreproducible, multimodal DLS traces indicative of size heterogeneity (Fig. 5B). These data confirm previous studies that show lyophilization causes liposome instability.^19^ In contrast, DLS analysis of LipoCatch alone, LipoCatch/DOPC (1 : 1), and LipoCatch/DOPC (1 : 4) nanostructures after lyophilization and rehydration revealed a major peak that closely overlapped with the pre-lyophilization trace (Fig. 5C–E, respectively). Although secondary populations were detected, these features were often reproducible across measurements, suggesting that the primary nanostructure and associated secondary assemblies remain relatively stable upon rehydration. However, LipoCatch/DOPC (1 : 8) exhibited less reproducible size distributions after rehydration, with increased variability between measurements, consistent with partial aggregation induced by lyophilization (Fig. 5F). Together, these data show that LipoCatch alone and LipoCatch/DOPC formulations at molar ratios of 1 : 1 and 1 : 4 exhibit greater tolerance to lyophilization compared to conventional DOPC liposomes alone.

## Discussion

Here, we established LipoCatch as a modular biomaterial for the formation of lipoprotein-based nanostructures. Key features of this material include its biosynthetic production, self-assembly, and incorporation of a SpyCatcher protein domain that allows for the versatile attachment of SpyTagged proteins. We demonstrate that detergent-solubilized LipoCatch forms nanostructures that are larger than non-lipidated SpyCatcher. We further show that when LipoCatch is covalently conjugated to SpyTag-MBP or SpyTag-GFP, it maintains its micelle-like morphology while increasing in diameter.

We also developed a protocol for incorporating lipids, representing the first demonstration of bacterial lipoprotein-based nanoparticles associating with exogenously added phospholipids. For lipid screening experiments, we selected four distinct lipids with identical acyl chains but different headgroup structures: DOPC, DOPE, DOPG, and PE CF. Each lipid is diacylated with unsaturated oleic acid (18 : 1), enabling formulation at room temperature. At specific molar ratios of LipoCatch : DOPC, the phospholipid content can be used to tune nanostructure size without extensive post-assembly sizing procedures. We also noted an increase in nanostructure %Pd with increasing equivalents of DOPC, which could be due to many factors, including less uniform DOPC incorporation at higher concentrations, the presence of DOPC only assemblies, or aggregation of nanostructures. TEM analysis of LipoCatch/DOPC nanostructures revealed solid, micelle-like particles with an irregular shape, rather than vesicular structures observed in liposome preparations. This non-spherical, micelle-like morphology could arise from nonuniform DOPC incorporation or irregular packing of non-spherical LipoCatch protein domains. Possibly, at higher lipid ratios vesicular assemblies with LipoCatch could be formed, although further experiments would be needed to confirm this.

DOPC, DOPE, and DOPG all formed homogeneous nanostructures with LipoCatch at a molar ratio of LipoCatch : phospholipid = 1 : 4, as determined by DLS. At a higher molar ratio of 1 : 20, DOPC, DOPE, and DOPG formed heterogeneous mixtures with LipoCatch. However, we also noted subtle differences in LipoCatch/phospholipid nanostructures based on headgroup variation. LipoCatch assembly with DOPE resulted in higher %Pd nanostructures than assembly with DOPC at a 1 : 4 molar ratio. This increase in %Pd could be caused by factors including the shape of the PE headgroup or its smaller size compared to the PC headgroup, possibly affecting interactions with LipoCatch monomers or with other DOPE lipids. DOPG resulted in larger assemblies with LipoCatch compared to DOPC and DOPE. Although not directly tested, this increase in size could possibly be due to electrostatic interactions between the negatively charged PG headgroup and LipoCatch, interactions between the DOPG lipids themselves, or a mixture between these factors. Future studies could explore this in more detail. In addition, the incorporation of a dye–lipid conjugate, PE CF, into LipoCatch nanostructures provides precedent for possible inclusion of other lipid-modified small molecules, including lipidated drugs. LipoCatch can therefore be easily modified both covalently and non-covalently. Finally, LipoCatch, LipoCatch/DOPC (1 : 1), and LipoCatch/DOPC (1 : 4) nanostructures have enhanced stability to lyophilization compared to DOPC liposomes. Together, these results establish LipoCatch as a flexible and robust platform for the assembly and functionalization of lipoprotein-based nanostructures.

Given that bacterial lipoprotein nanostructures represent a new class of biomaterial, below, we place their properties in context by comparison with established nanoparticle platforms, including liposomes, protein nanocages, lipopeptide nanoparticles, and outer membrane vesicles.

Liposomes are widely used as platforms for drug and vaccine delivery^15^ (Fig. S4A, panel 1). However, disadvantages include their instability to lyophilization and the multiple sizing steps needed to produce a uniform distribution of unilamellar liposomes.^15,18^ The LipoCatch/phospholipid nanostructures described in this work instead require no sizing procedures and demonstrate stability to lyophilization and rehydration. The ease of fabrication allows for rapid and reproducible assembly of monodisperse nanostructures, reducing technical complexity and equipment requirements relative to conventional liposome preparation. In addition, their resistance to lyophilization suggests potential utility in resource-limited settings where cold-chain infrastructure is unavailable.

Protein nanocages, such as ferritin and engineered particles (*e.g.* mi3), are self-assembling protein complexes that form well-defined, hollow structures (Fig. S4A, panel 2).^12,20,21^ Their assembly depends on precise protein–protein interactions at specific interfaces, making addition of an extra protein domain and cargo loading difficult.^22–24^ In contrast, protein-functionalization of LipoCatch with SpyTagged proteins (MBP and GFP) does not require prior knowledge of protein interfaces, simplifying modular modification.

Lipopeptides are composed of a short amino acid stretch linked to a hydrophobic lipid group.^25^ These materials self-assemble into various nanostructures such as micelles and wormlike structures (Fig. S4A, panel 3). However, lipopeptides are often produced through solid-phase synthesis, which limits scalability and ease of production for longer sequences.^3^ In contrast, bacterial lipoproteins can be produced biosynthetically using recombinant DNA technology and can incorporate full-length globular protein domains, enabling genetic encoding and a more straightforward production to impart diverse properties.

Outer membrane vesicles (OMVs) are nanosized phospholipid-based vesicles secreted by Gram-negative bacteria that have gained increasing interest as nanomedicine platforms including applications in vaccine development and drug delivery (Fig. S4A, panel 4).^26^ However, a key limitation of OMVs is the presence of lipopolysaccharide, an endotoxic component of Gram-negative bacteria, which is implicated in sepsis pathogenesis and raises safety concerns.^27^ In addition, OMVs are technically challenging to isolate, often requiring multistage differential centrifugation to separate vesicles based on size.^26^ In contrast, LipoCatch/phospholipid nanostructures are compositionally well-defined and can be prepared without such complex isolation protocols. Our work suggests that bacterial lipoprotein/phospholipid mixtures can be used to create defined OMV-like nanostructures.

Nanoparticle size is a critical parameter that influences biodistribution, cellular uptake, circulation time, and cargo loading capacity.^14^ Here, we show that, in contrast to existing nanoparticle platforms, LipoCatch size can be readily tuned by adjusting phospholipid content without altering protein sequence or requiring post-assembly processing, such as sonication or filtration using syringe filters with defined pore sizes. By comparison, the size of protein nanocages is fixed by protomer architecture, lipopeptide nanoparticles often require sequence redesign to modify size, and OMVs exhibit limited and indirect size control. This tunability represents a practical advantage of LipoCatch over established nanoparticle platforms.

In comparison to the nanostructures listed above, a key distinction lies in the immunogenicity of bacterial lipoproteins. Unlike the biomaterials listed above, bacterial lipoproteins are highly immunogenic.^28^ They contain a covalently attached lipid moiety that is classified as a pathogen associated molecular pattern that functions as a Toll-like receptor 2 ligand, enabling direct activation of human innate immune receptors.^29^ Thus, the intrinsic immunostimulatory property makes bacterial-lipoprotein-based nanostructures well-suited for applications that benefit from enhanced immune activation, such as vaccine formulations, while simultaneously limiting their applicability for systemic delivery or for therapeutic applications that require repeated dosing.

Despite the advantages demonstrated here, several aspects of this technology remain unknown and warrant further investigation. These include defining the size and oligomeric state limitations of SpyTagged proteins that can be displayed on LipoCatch without perturbing nanostructure assembly. In addition, the behavior and stability of lipoprotein nanostructures under detergent-free conditions remain unclear. The number of LipoCatch monomers per nanostructure has not yet been elucidated, nor has the number of lipids associated to each nanostructure. Future quantitative work could focus on determining the bacterial lipoprotein-to-lipid ratio within these nanostructures. Such measurements could help establish how composition influences size and morphology. For nanostructure characterization, DLS and negative stain TEM were used to analyze particle size and morphology. While these techniques provide valuable information regarding average size and morphology, they are limited to two-dimensional structural characterization. Future studies could incorporate techniques such as electron tomography to provide additional three-dimensional structural information. Finally, the principles governing how different classes of associated lipids, including glycolipids, neutral lipids (*e.g.* cholesterol), and ether-linked lipids influence nanostructure assembly and stability have yet to be established. In particular, studies examining acyl chain saturation and acyl chain length would be of interest. Variation in the lipid properties are known to alter lipid packing, membrane fluidity, and phase behavior, which may in turn influence how effectively exogenous lipids are incorporated into bacterial lipoprotein nanostructures. Understanding the rules governing their assembly could provide valuable insight into the design potential of the biomaterial.

During the course of this work, another group reported the lipidation of SpyCatcher using the biosynthetic bacterial lipidation pathway, its conjugation to a SpyTagged protein, and its use in a vaccine formulation.^30^ However, several key differences distinguish this recent report from the present study. First, we employed the signal peptide from NmMetQ of *N. meningitidis*, whereas the recent report used a signal peptide from *E. coli*. Second, they employed a different purification strategy involving alternative detergents. As a result, the final formulations differ, as the detergent was removed in their final purification step, whereas it was retained in ours. Importantly, the assembly of lipidated SpyCatcher with exogenously added lipids was not explored in the referenced work, nor was the stability of the nanostructures to lyophilization assessed. Despite these differences, their lipidated SpyCatcher and our LipoCatch nanoparticles were of comparable size and morphology, as determined by negative-stain TEM, suggesting that lipidated SpyCatcher represents a robust protein for nanoparticle formation across distinct construct designs and purification strategies.

In summary, we developed LipoCatch, a bacterial lipoprotein-based biomaterial that can conjugate covalently to SpyTagged proteins, and established a protocol for the non-covalent incorporation of exogenously added lipids. Moreover, we show that these nanostructures are more tolerant to lyophilization than DOPC liposomes, highlighting a practical advantage over conventional phospholipid-based nanoparticles. Together, this work positions bacterial lipoproteins as a distinct and robust class of biomaterial for future nanotechnology applications.

## Conclusion

In comparison to existing technologies, a biomaterial that uses bacterial lipoproteins as a building block could offer three key advantages: (1) the ability to self-assemble into nanosized micelle-like structures without complex manipulations, (2) customizability through recombinant DNA technology to impart novel functions to the protein domain, and (3) cost-effective production using the *E. coli* expression system. Their unique composition of a protein domain and a covalently attached lipid moiety sets them apart from established nanostructures made from other biological materials, such as liposomes, and protein-only nanomaterials. Overall, this work makes progress towards a fundamental understanding of the assembly of bacterial lipoproteins into nanostructures and their interactions with other molecules.

## Materials and methods

### Materials

DDM was purchased from Anatrace and stored at −20 °C until use. DOPC, DOPE, DOPG, and PE CF were purchased from Avanti Polar Lipids and stored in chloroform solution at −20 °C until use. SEC gel filtration standards were purchased from Bio-Rad (Catalog #1511901).

### Protein expression and purification

The amino acid sequences of SpyCatcher003 (GenBank MN433887), SpyTag003,^8^ MBP (UniProt P0AEX9), GFP (variant derived from GenBank ASL68970), and *N. meningitidis* MetQ signal peptide (UniProt Q7DD63) were obtained from public databases or published sources. To aid in purification, a decahistidine (10H) was also added to the C-terminus of the protein-encoding genes (Fig. S2). The DNA sequences of LipoCatch, non-lipidated SpyCatcher, SpyTag, MBP, and GFP were created using the GenSmart™ Codon Optimization tool (GenScript). These sequences were inserted into a pET-21b(+) ampicillin-resistant vector between NdeI and XhoI sites, under the control of a T7 promoter (GenScript). All proteins were expressed as previously described.^1^ Briefly, proteins were expressed in *E. coli* BL21 (DE3) gold cells (Agilent) using ZYM-5052 autoinduction media containing 100 mg L^−1^ ampicillin at 30 °C for 30 h. Cells were harvested by centrifugation at 4785 RCF (JLA 8.1; 5000 rpm; Beckman Coulter) for 15 min and the cell paste was flash frozen in liquid nitrogen for storage at −80 °C.

To purify LipoCatch and non-lipidated SpyCatcher proteins, ∼10 g of cell paste was thawed at room temperature (RT), then placed in a 100 mL solution of 25 mM Tris HCl pH 7.5, 100 mM NaCl, 40 mg of lysozyme, 4 mg of DNase, and one cOmplete protease inhibitor cocktail tablet (Sigma-Aldrich). Cells were lysed by the addition of 1% w/v DDM and by stirring the homogenate for 3 h at 4 °C. Cell debris was removed by centrifugation at 94 834 RCF (Ti45; 35 000 rpm; Beckman Coulter) for 45 min. Imidazole was then added to the lysate to a final concentration of 70 mM to remove nonspecific protein binders during affinity purification.

Proteins were purified using a 5 mL HisTrap HP column (Cytiva) pre-equilibrated with 25 mM Tris HCl pH 7.5, 100 mM NaCl, and 0.05% w/v DDM, then eluted with a solution of 25 mM Tris HCl pH 7.5, 100 mM NaCl, and 300 mM imidazole. The eluted proteins were then loaded onto a Hiload 16/600 Superdex 200 pg (Cytiva) SEC column pre-equilibrated with 25 mM Tris HCl pH 7.5, 100 mM NaCl, and 0.05% w/v DDM. For all proteins, the peak fractions were combined, frozen in liquid nitrogen, and stored at −80 °C until thawed.

### LC-MS analysis

Intact mass analysis of SpyCatcher and non-lipidated SpyCatcher proteins was performed using LC-MS, as previously described.^1^ Briefly, protein masses were determined by ultra-performance liquid chromatography-mass spectrometry. Specifically, the proteins were analyzed by LC-MS on the Waters Acquity UPLC and Thermo Exploris 240 Orbitrap BioPharma mass spectrometer. Proteins dissolved in buffer (25 mM Tris HCl pH 7.5, 100 mM NaCl, 0.05% w/v DDM) were injected onto a Waters BioResolve RP mAb polyphenyl 450 A 2.7 u 2.1 × 100 mm column maintained at 80 °C and flow rate 0.2 mL min^−1^. The injection volume was 3 µL. Chromatographic separation occurred over the course of 20 minutes. This separation utilized a gradient starting at 95% solvent A (0.1% formic in water) and 5% solvent B (0.1% formic acid in acetonitrile), arriving at 20% solvent A and 80% solvent B at 9.5 min, and ending with 95% solvent A and 5% solvent B at 20 min.

Mass spectra were collected in positive full scan MS mode, Orbitrap resolution 120 000, mass range 500–4000 Da, RF lens 60 V, and source fragmentation energy 15 V. Deconvoluted mass spectra were generated with the Byos Intact software (v5.7.45-g365c760c8dx64) from Protein Metrics. Graphs of these spectra were then generated in MATLAB (vR2023b) and imported into Adobe Illustrator (2024) to modify the text and font to increase readability.

### Preparation of LipoCatch nanostructures

LipoCatch nanostructures were prepared by centrifugal ultrafiltration of detergent purified protein. Centrifugation was performed using an Amicon Ultra-0.5 centrifugal filter with a molecular weight cutoff of 3000 Da. Before using, the filters were hydrated with DI water. Detergent-purified LipoCatch (200 µL) was mixed with buffer without detergent (200 µL, 25 mM Tris HCl pH 7.5, 100 mM NaCl, no DDM), and centrifuged at 9391 RCF (FA-24x2; 10 000 rpm; Eppendorf) for 10 min. This process was repeated a total of four times. The solution was then analyzed immediately by UV/Vis to determine protein concentration and DLS to determine particle size and distribution.

### Preparation of LipoCatch : SpyTag nanostructures

LipoCatch (2.83 mg mL^−1^, 0.003 µmol, 17.6 µL) was combined with SpyTag-GFP (0.53 mg mL^−1^, 0.003 µmol, 175.4 µL) or SpyTag-MBP (1.36 mg mL^−1^, 0.003 µmol, 103 µL), and left to react at room temperature for 3 h. LipoCatch : SpyTag-MBP (100 µL) or LipoCatch : SpyTag-GFP (190 µL) conjugate was combined with 300 µL or 260 µL, respectively, of buffer without detergent (25 mM Tris HCl pH 7.5, 100 mM NaCl, no DDM). The samples were concentrated to 200 µL using an Amicon Ultra-0.5 centrifugal filter with a molecular weight cutoff of 10 000 Da at 9391 RCF (FA-24x2; 10 000 rpm; Eppendorf) for 5 min.

### Preparation of LipoCatch/phospholipid nanostructures

LipoCatch/phospholipid nanostructures were prepared according to previously described procedures for lipid thin-film hydration, with modifications.^15^ Briefly, phospholipids were dissolved in chloroform at concentrations ranging from 1–5 mg mL^−1^ and concentrated under a flow of N_2_ to produce a lipid thin film. The film was further dried in a vacuum desiccator for 30 min. Purified bacterial lipoprotein (1.5 mg mL^−1^, 0.018 µmol, 200 µL) in buffer with detergent (25 mM Tris HCl pH 7.5, 100 mM NaCl, 0.05% w/v DDM) was then used to resuspend the lipid film and form nanostructures. In control samples with DOPC, DOPE, or DOPG alone, buffer with the same detergent concentration was used to hydrate films rather than detergent purified bacterial lipoprotein solution. The thin films were briefly agitated with a plastic pipette tip to aid resuspension. The solution was then equilibrated with gentle shaking at room temperature for 1 h. An ultrafiltration centrifugation step was then performed using an Amicon Ultra-0.5 centrifugal filter with a molecular weight cutoff of 3000 Da. Before using, the centrifugal filters were hydrated with DI water. LipoCatch/phospholipid solutions (200 µL) were mixed with buffer without detergent (200 µL, 25 mM Tris HCl pH 7.5, 100 mM NaCl, no DDM), and concentrated to 200 µL at 9391 RCF (FA-24x2; 10 000 rpm; Eppendorf) for 10 min. This process was repeated a total of four times. The solution was then analyzed immediately by UV/Vis to determine protein concentration and DLS to determine particle size and distribution.

### Preparation of LipoCatch/PE CF nanostructures

PE CF nanostructures were prepared using the modified thin-film hydration procedure described for LipoCatch/DOPC nanostructure preparation. PE CF (1 mg mL^−1^, 0.02 µmol) in chloroform solution was added to a glass vial and solvent was removed by N_2_ flow. PE CF films were then dried in a vacuum desiccator for 30 min. Next, solutions of LipoCatch (0.02 µmol, 400 µL), SpyCatcher (0.02 µmol, 400 µL), buffer without detergent (25 mM Tris HCl pH 7.5, 100 mM NaCl, 200 µL), or buffer with detergent (25 mM Tris HCl pH 7.5, 100 mM NaCl, 0.05% w/v DDM, 200 µL) were added to the PE CF films. The films were agitated with a plastic pipette tip to aid in resuspension, followed by equilibration with gentle shaking for 30 min. Resuspended PE CF solution (200 µL) was combined with buffer without detergent (200 µL, 25 mM Tris HCl pH 7.5, 100 mM NaCl, no DDM), and concentrated to 200 µL at 9391 RCF (FA-24x2; 10 000 rpm; Eppendorf) for 10 min. This process was repeated a total of four times. The solution was then analyzed immediately by UV/Vis, fluorescence spectroscopy, and DLS.

### Negative stain TEM and dynamic light scattering

Protein solution was diluted to a final concentration of 0.01 mg mL^−1^ using buffer without detergent (25 mM Tris HCl pH 7.5, 100 mM NaCl, no DDM), adsorbed onto glow-discharged copper grids (Formvar/Carbon 200 mesh; Ted Pella, Inc.) for 3 min, and then blotted using dry filter paper. The sample was stained by placing the grid against the surface of a 10 µL drop of 1% uranyl acetate (Electron Microscopy Sciences) on parafilm four times, blotting immediately after the first three drops and allowing the fourth drop to rest on the grid for 1 min prior to blotting.

Then, the grid was left on filter paper to fully dry. Micrographs were obtained using an FEI Titan environmental transmission electron microscope operated at an acceleration voltage of 300 kV and an actual magnification of 57 000×, equipped with a Gatan K3 camera. ImageJ 3 was used to process TEM micrographs. A bandpass filter was applied (large structures filtered up to 100 pixels, small structures filtered down to 3 pixels), followed by brightness and contrast adjustment.

Dynamic light scattering measurements were performed using the default Measure Size option on a DynaPro NanoStar II (Wyatt Technology Corporation) instrument. A total of three measurements per sample were acquired. Hydrodynamic diameter and %Pd values calculated using the cumulants algorithm specified in ISO 22412 and ASTM 2490-09 were then exported directly from the Wyatt DYNAMICS software. Microsoft Excel was used to calculate the mean and standard deviation of three technical replicates. MATLAB (vR2023b) was then used to plot the triplicate or representative DLS traces using spline interpolation. In our analysis, we considered %Pd values >30 to reflect polydisperse particle distributions.

### Absorbance and fluorescence spectroscopy

Fluorescence spectra were collected using a BioTek Synergy H1 microplate reader. Fluorescence emission intensities were measured with an excitation/emission of 485 nm (bandwidth 12.5 nm)/528 nm (bandwidth 12.5 nm) at a read height of 7 nm. Fluorescence emission spectra were collected at an excitation of 470 nm and an emission range of 500 nm to 650 nm with an emission step of 2 nm and a read height of 7 nm.

### Lyophilization stability assay

DOPC-only liposome controls were prepared using a thin-film hydration method followed by extrusion. DOPC (25 mg mL^−1^, 0.33 µmol, 10.5 µL) in chloroform solution was added to a glass vial and solvent was removed by N_2_ flow. DOPC films were then dried in a vacuum desiccator for 30 min. Next, buffer without DDM (922 µL, 25 mM Tris HCl pH 7.5, 100 mM NaCl, no DDM) was added to the DOPC films. The films were agitated with a plastic pipette tip to aid in resuspension, followed by equilibration with gentle shaking for 30 min. Next, the lipid suspensions were extruded 10 times using a mini extruder (Avanti Polar Lipids) with a Whatman™ Nuclepore™ track-etched membrane filter (PCTE, 0.1 µm, 19 mm). LipoCatch/DOPC nanostructures were prepared using the modified-thin film hyration procedure previously described, without extrusion steps. Aliquots of LipoCatch or LipoCatch/DOPC nanostructures (100 µL, 1 mg mL^−1^) were added to PCR tubes and flash-frozen in liquid nitrogen. The samples were freeze-dried for 24 h at 0.04 mBar at −87 °C using a Labconco 6L lyophilizer. The samples then reconstituted in 100 µL DI water and analyzed immediately by DLS. Next, the samples were centrifuged at 15 871 RCF (FA-24x2; 13 000 rpm; Eppendorf) for 10 min to remove any aggregated protein before analysis by SDS-PAGE with Coomassie staining. All SDS PAGE analyses were performed using Any kD™ Criterion™ TGX Stain-Free™ Protein Gels (Catalog #5678124) with Precision Plus Protein Dual Color Standards (Catalog #1610374). Gel densitometry analysis was conducted using ImageJ 3. Microsoft Excel was used to calculate the mean and standard deviation of three technical replicates. MATLAB (vR2023b) was then used to plot the average of triplicate measurements.

### Statistical analysis

For DLS analysis, mean and standard deviation of *D*_h_ and average %Pd of three technical replicates were calculated in Microsoft Excel using the raw data provided by the Wyatt DYNAMICS software. For the relationship between mean *D*_h_ and DOPC equivalents, linear regression was performed in MATLAB (vR2023b). The mean and standard deviation of PE CF fluorescence intensity values were calculated and plotted in MATLAB (vR2023b), with the standard deviation as error bars. For gel densitometry analysis, the band intensity values were measured using ImageJ 3, and the mean and standard deviation were calculated and plotted in MATLAB (vR2023b), with the standard deviation as error bars.

### Model building

A structural prediction of the globular domain of LipoCatch was generated using the AlphaFold 3 Server.^31^ The amino acid sequence of SpyCatcher003 (lacking the signal peptide and decahistidine tag) was used as the input, and the predicted fold with the highest confidence score was used for further model building. Acyl chain structures (palmitic acid, 16 : 0) were generated using ChemDraw (v23.1.1) and energy-minimized using Open Babel (v3.1.1) *via* the obminimize function. The minimized acyl chains were then appended to the N-terminal cysteine of the LipoCatch globular domain *via* its sulfur atom (*S*-diacylation) and its α-amino group (*N*-acylation) using ChimeraX (v1.7.1).

## Author contributions

F. A. S., C. J. S, M. A. A., and N. G. S. methodology; F. A. S., C. J. S, M. A. A., and N. G. S. conceptualization; F. A. S., C. J. S., and N. G. S. visualization; F. A. S., C. J. S., and N. G. S. writing – original draft; F. A. S., C. J. S., M. A. A., M. A. T., S. A. W., and N. G. S. investigation; F. A. S., C. J. S., M. A. A., and N. G. S. formal analysis; N. G. S. supervision; N. G. S. funding acquisition; F. A. S., C. J. S., and N. G. S. writing – review and editing.

## Conflicts of interest

The authors declare that they have no conflicts of interest with the contents of this article.

## Abbreviations

DLS: Dynamic light scattering
SEC: Size-exclusion chromatography
LC-MS: Liquid chromatography mass spectrometry
UPLC-MS: Ultra-performance liquid chromatography-mass spectrometry
DDM: *n*-Dodecyl-β-d-maltoside
LC-MS: Liquid chromatography-mass spectrometry
TEM: Transmission electron microscopy
MBP: Maltose binding protein
GFP: Green fluorescent protein
DOPC: 1,2-Dioleoyl-*sn*-glycero-3-phosphocholine
DOPE: 1,2-Dioleoyl-*sn*-glycero-3-phosphoethanolamine
DOPG: 1,2-Dioleoyl-*sn*-glycero-3-phosphoglycerol
PE CF: 1,2-Dioleoyl-*sn*-glycero-3-phosphoethanolamine-*N*-(carboxyfluorescein)

## Supplementary Material

NA-008-D6NA00554C-s001

## Data Availability

Data supporting the findings of this study are available on request from the corresponding author. Supplementary information (SI): LC-MS data and protein sequence information. See DOI: https://doi.org/10.1039/d6na00554c.
