# Supplementary material for: Development and characterization of LipoCatch: a bacterial lipoprotein-based biomaterial that self-assembles into nanostructures
Source: Nanoscale Adv. 2026 Jul 9;8(16):4605–18. doi: 10.1039/d6na00554c (PMC13394007; doi:10.1039/d6na00554c)
Supplement: Supplementary file 1 [file NA-008-D6NA00554C-s001.pdf]

Supplementary Information (SI) for Nanoscale Advances.
This journal is © The Royal Society of Chemistry 2026

Supplementary Information

Development and characterization of LipoCatch: a bacterial lipoprotein-based biomaterial that self-assembles into nanostructures

Francesca Starvaggi[1], Claire Stewart[1], Marc A. Arslanian[1], Matthew A. Trevin˜o[1], Scott Weston[1], and Naima G. Sharaf[1,*]

[1]*Department of Biology, Stanford University, Stanford CA 94305, USA*

[1] *Department of Chemistry, Stanford University, Stanford CA 94305, USA*

[*]To whom correspondence should be addressed

[*]E-mail: ngsharaf@stanford.edu

A. LipoSpyCatcher (LipoCatch)>

MKTFFKTLSAAALALILAACGSGGSGGSGMVTTLSGLSGEQGPSGDMTTEEDSATHIKFSKRDEDGRELAG
ATMELRDSSGKTISTWISDGHVKDFYLYPGKYTFVETAAPDGYEVATPIEFTVNEDGQVTVDGEATEGDAHTG
SGSGHHHHHHHHHH*

B. Non lipidated SpyCatcher>

MVTTLSGLSGEQGPSGDMTTEEDSATHIKFSKRDEDGRELAGATMELRDSSGKTISTWISDGHVKDFYLYPG
KYTFVETAAPDGYEVATPIEFTVNEDGQVTVDGEATEGDAHTGSGSGHHHHHHHHHH*

C. SpyTag-MBP>

MKIKTGARILALSALTTMMFSASALAKIEEGKLVIWINGDKGYNGLAEVGKKFEKDTGIKVTVEHPDKLEEKFP
QVAATGDGPDIIFWAHDRFGGYAQSGLLAEITPDKAFQDKLYPFTWDAVRYNGKLIAYPIAVEALSLIYNKDLLP
NPPKTWEEIPALDKELKAKGKSALMFNLQEPYFTWPLIAADGGYAFKYENGKYDIKDVGVDNAGAKAGLTFLV
DLIKNKHMNADTDYSIAEAAFNKGETAMTINGPWAWSNIDTSKVNYGVTVLPTFKGQPSKPFVGVLSAGINAA
SPNKELAKEFLENYLLTDEGLEAVNKDKPLGAVALKSYEEELAKDPRIAATMENAQKGEIMPNIPQMSAFWYA
VRTAVINAASGRQTVDEALKDAQTRITKGSGGSGGSGRGVPHIVMVDAYKRYKHHHHHH*

D. SpyTag-GFP>

MGRGVPHIVMVDAYKRYKSGGGSGRKGEELFTGVVPILVELDGDVNGHKFSVRGEGEGDATNGKLTLKFIC
TTGKLPVPWPTLVTTLTYGVQCFARYPDHMKQHDFFKSAMPEGYVQERTISFKDDGTYKTRAEVKFEGDTL
VNRIELKGIDFKEDGNILGHKLEYNFNSHNVYITADKQKNGIKANFKIRHNVEDGSVQLADHYQQNTPIGDGP
VLLPDNHYLSTQSVLSKDPNEKRDHMVLLEFVTAAGITHGMDELYKGSGSGSHHHHHHHHHH*

Figure S1: Construct design. Sequence of **A.** LipoSpyCatcher (LipoCatch), **B.** non-lipidated SpyCatcher, **C.** SpyTag-MBP, and **D.** SpyTag-GFP. Signal sequences, GS linker sequences, protein globular domain encoding sequences, and polyhistidine tag sequences are shown in green, purple, black, and orange, respectively.

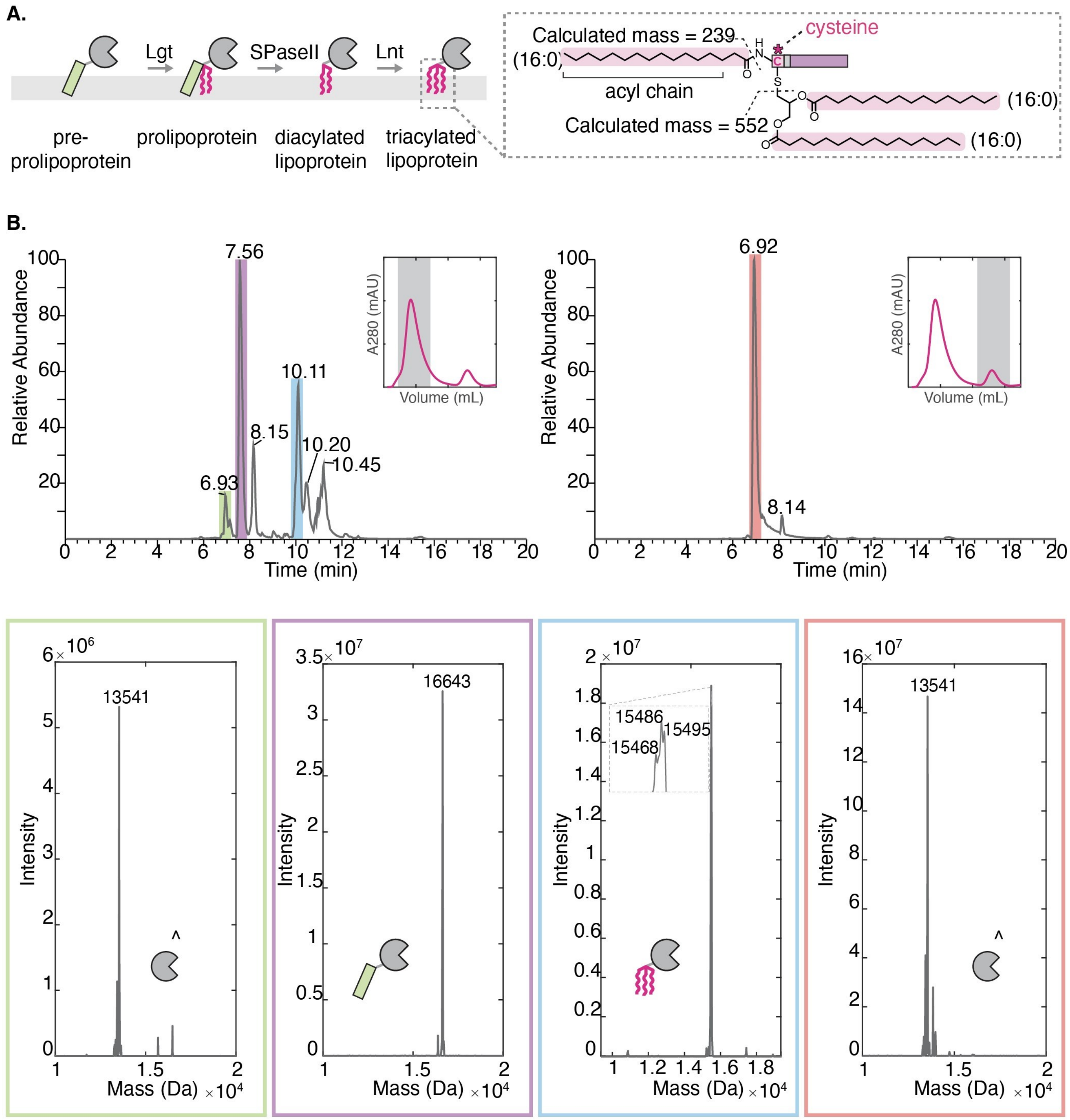

Figure S2: LC/MS of LipoCatch. A. Diagram of the canonical bacterial lipoprotein maturation pathway. **B.** Total ion chromatogram (TIC) of the major and minor SEC peak fractions, with the corresponding deconvoluted mass spectra of the highlighted peaks at various retention times. Inset is the LipoCatch SEC trace. Some peaks not

highlighted from both TICs, (RT = 8.15, 10.20, and 8.14 min) had single ion species of approximately 1020 Da. The remaining peak (RT = 10.45 min), contained free lipids.

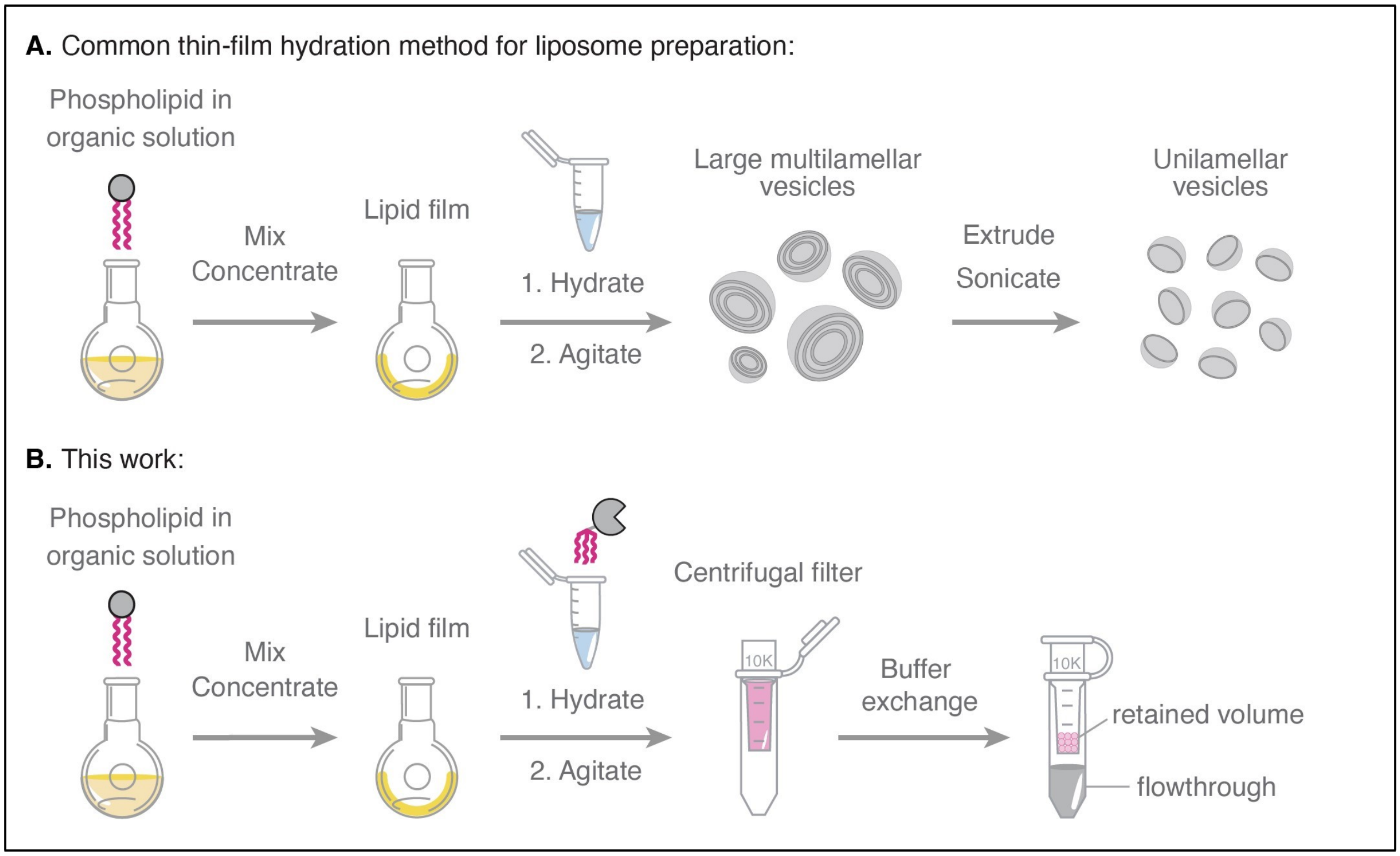

Figure S3: Nanostructure formation using a thin-film hydration method. A. Schematic representation of a typical thin-film hydration method for liposome formation. **B.** Proposed procedure for formation of LipoCatch/phospholipid nanostructures, using a modified thin-film hydration method.

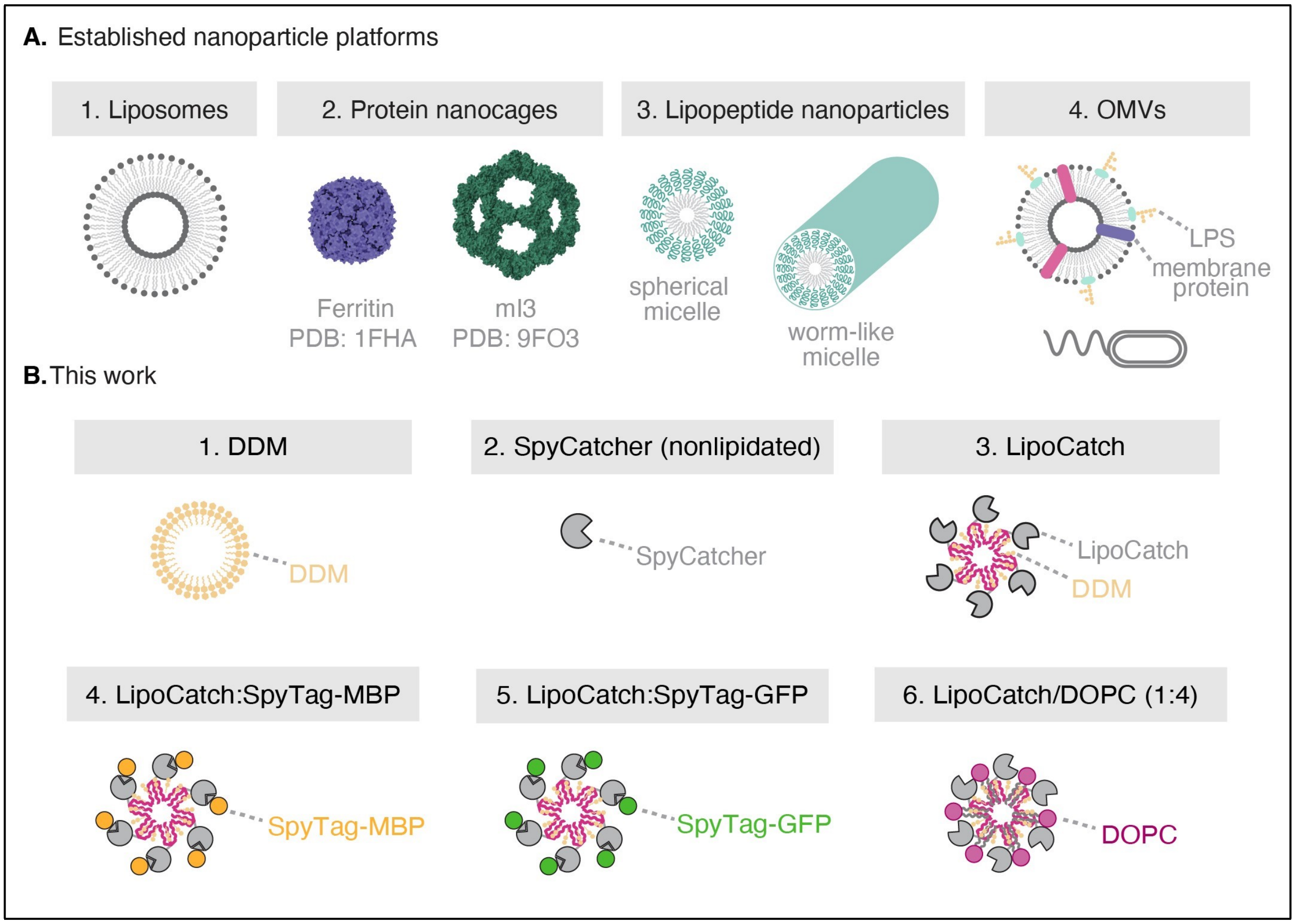

Figure S4: Cartoon models of nanostructures. A. Cartoon representation of select established nanostructures. **B.** Cartoon representation of DDM, SpyCatcher and nanostructures developed in this work.
